# Supplementary material for: Structure and biochemistry-guided engineering of an all-RNA system for DNA insertion with R2 retrotransposons
Source: Nat Commun. 2025 Jul 2;16:6079. doi: 10.1038/s41467-025-61321-z (PMC12222951; doi:10.1038/s41467-025-61321-z)
Supplement: Supplementary file 1 — Supplementary Information [file 41467_2025_61321_MOESM1_ESM.pdf]

Supplementary Information for

Structure and biochemistry-guided engineering of an all-RNA system for DNA insertion with R2 retrotransposons

KeHuan K. Edmonds, Max E. Wilkinson, Daniel Strebinger, Hongyu Chen, Blake Lash, Clarissa C. Schaefer, Shiyou Zhu, Dangliang Liu, Shai Zilberzwige-Tal, Alim Ladha, Michelle L. Walsh, Chris J. Frangieh, Nicholas A. Vaz Reay, Rhiannon K. Macrae, Xiao Wang & Feng Zhang[#]

[#]Correspondence: zhang@broadinstitute.org

This PDF file includes:

Supplementary Figs. 1-11

Other Supplementary Information files include the following:

Supplementary Data 1. Plasmid, oligonucleotide, and crRNA sequences used in this study.
Supplementary Data 2. Cryo-EM data collection, refinement, and validation statistics.
Supplementary Data 3. Off-target loci detected by the TTISS experiment.

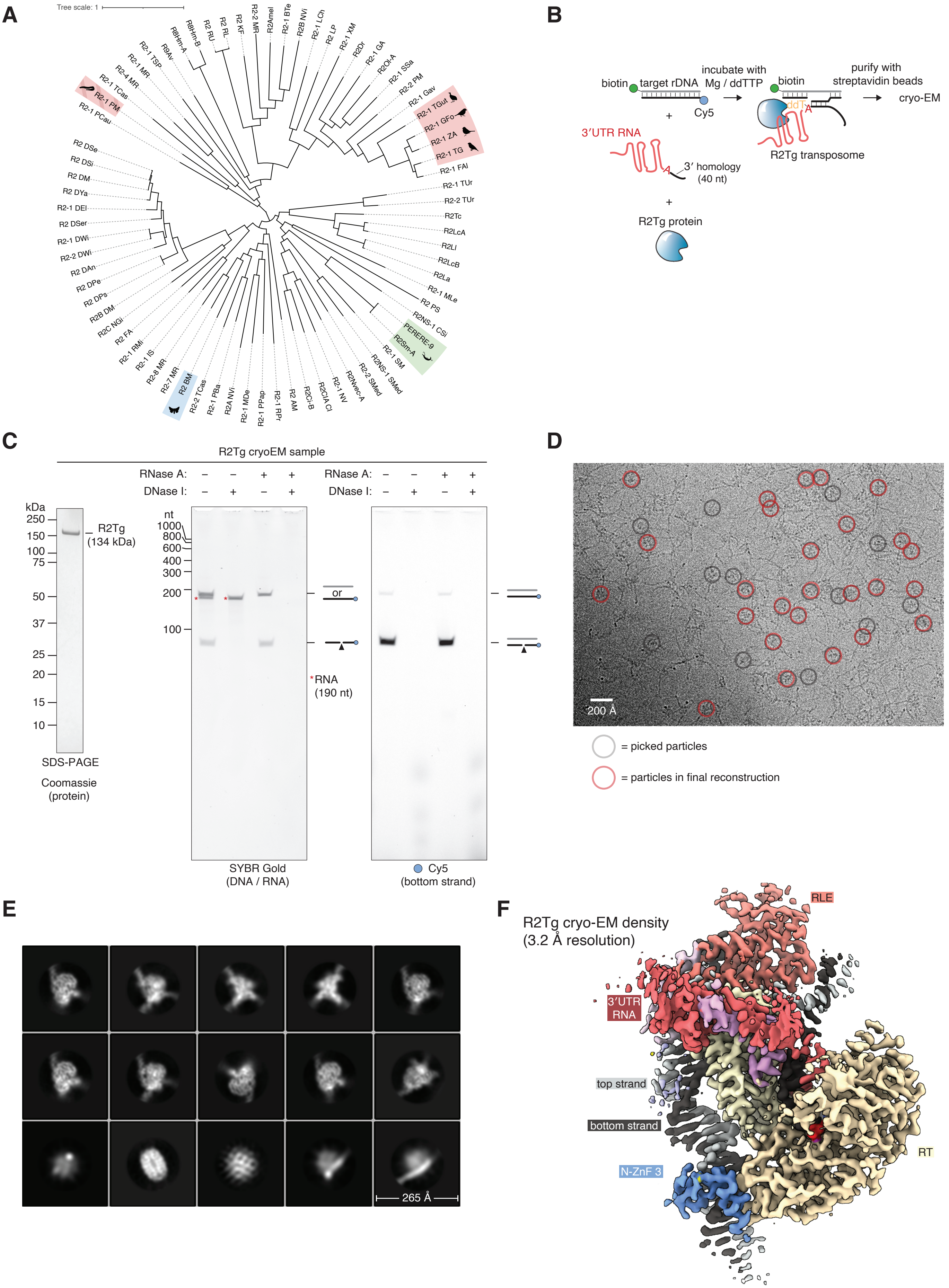
A
Tree scale: 1
R2-1 PM
R2-1 PCau
R2-1 TCas
R2-4 MR
R2-1 MR
R2-1 TSP
R9Av
R8Hm-A
R8Hm-B
R2 RU
R2 RL
R2 KF
R2-2 MR
R2Amel
R2-1 BTe
R2B NVi
R2-1 LCh
R2 LP
R2-1 XM
R2Dr
R2-1 GA
R2Ol-A
R2-1 SSa
R2-2 PM
R2-1 Gav
R2-1 TGut
R2-1 GFo
R2-1 ZA
R2-1 TG
R2-1 FAl
R2-1 TUr
R2-2 TUr
R2Tc
R2LcA
R2Ll
R2LcB
R2La
R2-1 MLe
R2 PS
R2NS-1 CSi
PERERE-9
R2Sm-A
R2-1 SM
R2NS-1 SMed
R2-2 SMed
R2Nvec-A
R2-1 NV
R2CIA CI
R2Ci-B
R2 AM
R2-1 RPr
R2-1 PPap
R2-1 MDe
R2A NVi
R2-1 PBa
R2-2 TCas
R2 BM
R2-7 MR
R2-8 MR
R2-1 IS
R2-1 RMi
R2 FA
R2C NGi
R2B DM
R2 DPs
R2 DPe
R2 DAn
R2-2 DWi
R2-1 DWi
R2 DSer
R2-1 DEl
R2 DYa
R2 DM
R2 DSi
R2 DSe
B
biotin
target rDNA
Cy5
+
3′UTR RNA
3′ homology (40 nt)
+
R2Tg protein
incubate with Mg / ddTTP
biotin
ddT
A
R2Tg transposome
purify with streptavidin beads
cryo-EM
C
R2Tg cryoEM sample
kDa
250
150
100
75
50
37
25
20
15
10
R2Tg (134 kDa)
SDS-PAGE
Coomassie (protein)
RNase A: – – + +
DNase I: – + – +
nt
1000
800
600
400
300
200
100
or
*RNA (190 nt)
SYBR Gold (DNA / RNA)
RNase A: – – + +
DNase I: – + – +
Cy5 (bottom strand)
D
200 Å
= picked particles
= particles in final reconstruction
E
265 Å
F
R2Tg cryo-EM density (3.2 Å resolution)
RLE
3′UTR RNA
top strand
bottom strand
N-ZnF 3
RT

Supplementary Fig. 1: Purification and cryo-EM analysis of the R2Tg TPRT complex.

A. R2 tree generated from ORF alignment. Orthologs tested in Fig. 1b are highlighted by clade.
B. Strategy for purifying the R2Tg TPRT complex
C. Analysis of the purified R2Tg TPRT complex, run once. For the left gel, the sample was directly applied to SDS-PAGE. For the right two gels, the sample was extracted with phenol-chloroform and ethanol precipitated first, before nuclease treatment as indicated and running on denaturing TBE-Urea PAGE. The same gel was visualized by SYBR Gold staining (middle) and by Cy5 fluorescence (right).
D. Example cryo-EM micrograph of the R2Tg TPRT complex.
E. Some example 2D class averages from all picked particles for the R2Tg TPRT complex.
F. Final cryo-EM density of the R2Tg TPRT complex.

Source data are provided as a Source Data file.

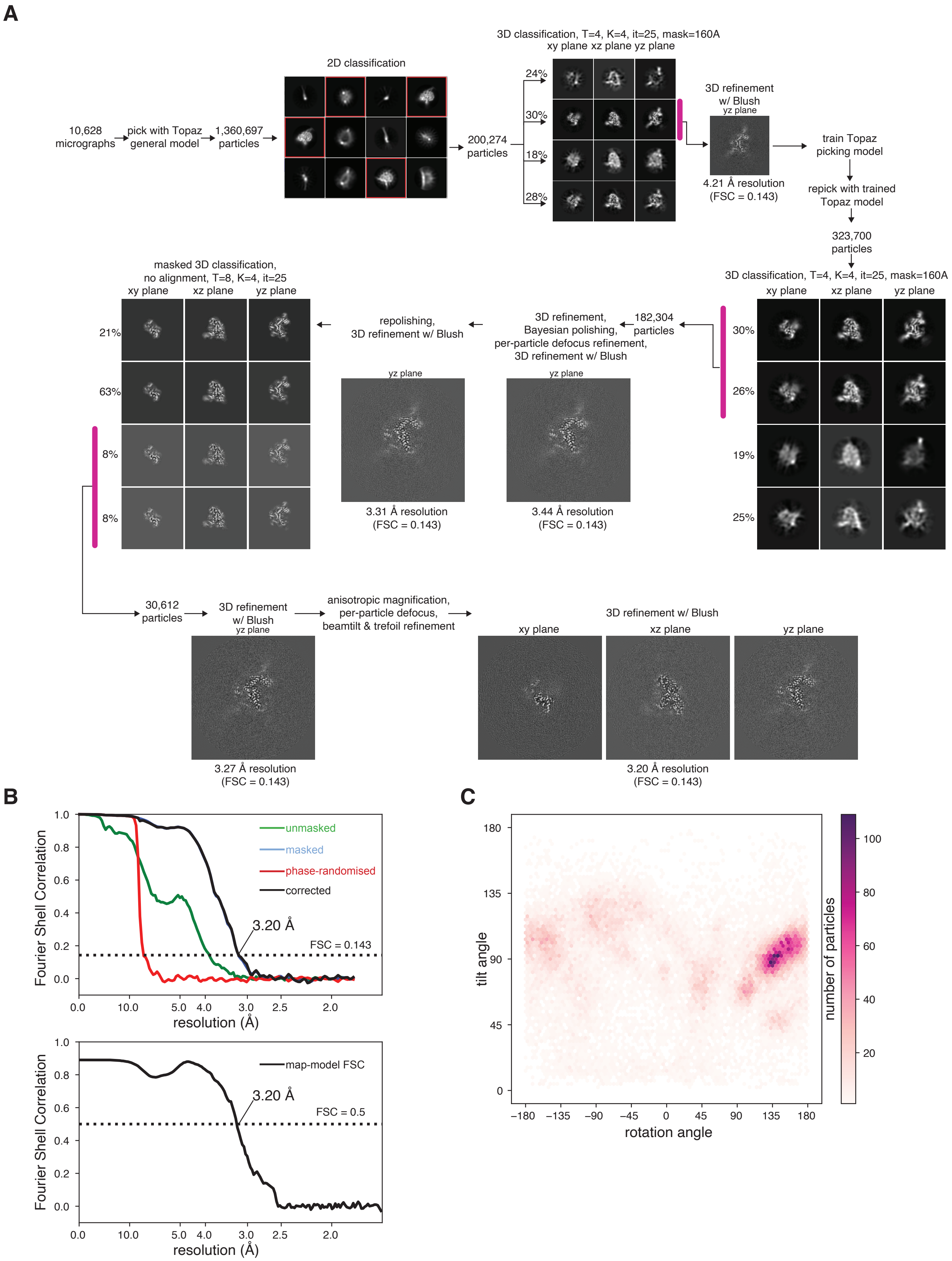
A
2D classification
3D classification, T=4, K=4, it=25, mask=160A
xy plane xz plane yz plane
24%
30%
18%
28%
3D refinement
w/ Blush
yz plane
4.21 Å resolution
(FSC = 0.143)
10,628
micrographs
pick with Topaz
general model
1,360,697
particles
200,274
particles
train Topaz
picking model
repick with trained
Topaz model
323,700
particles
3D classification, T=4, K=4, it=25, mask=160A
xy plane
xz plane
yz plane
30%
26%
19%
25%
182,304
particles
3D refinement,
Bayesian polishing,
per-particle defocus refinement,
3D refinement w/ Blush
yz plane
3.44 Å resolution
(FSC = 0.143)
repolishing,
3D refinement w/ Blush
yz plane
3.31 Å resolution
(FSC = 0.143)
masked 3D classification,
no alignment, T=8, K=4, it=25
xy plane
xz plane
yz plane
21%
63%
8%
8%
30,612
particles
3D refinement
w/ Blush
yz plane
3.27 Å resolution
(FSC = 0.143)
anisotropic magnification,
per-particle defocus,
beamtilt & trefoil refinement
3D refinement w/ Blush
xy plane
xz plane
yz plane
3.20 Å resolution
(FSC = 0.143)
B
Fourier Shell Correlation
unmasked
masked
phase-randomised
corrected
3.20 Å
FSC = 0.143
resolution (Å)
0.0
10.0
5.0
4.0
3.0
2.5
2.0
map-model FSC
3.20 Å
FSC = 0.5
C
tilt angle
rotation angle
number of particles
180
135
90
45
0
−180
−135
−90
−45
45
135
100
80
60
40
20

Supplementary Fig. 2: R2Tg cryo-EM data processing.

A. Flowchart outlining the cryo-EM data processing workflow. All data were processed in RELION. For 3D classifications, Relion options are specified, and three central slices are shown for each class.
B. Gold-standard Fourier Shell Correlation curves for the final reconstruction (Top). Map-to-model Fourier Shell Correlation as calculated in Phenix, softly masking the map around the fitted model.
C. Orientation distribution plot for the final reconstruction.

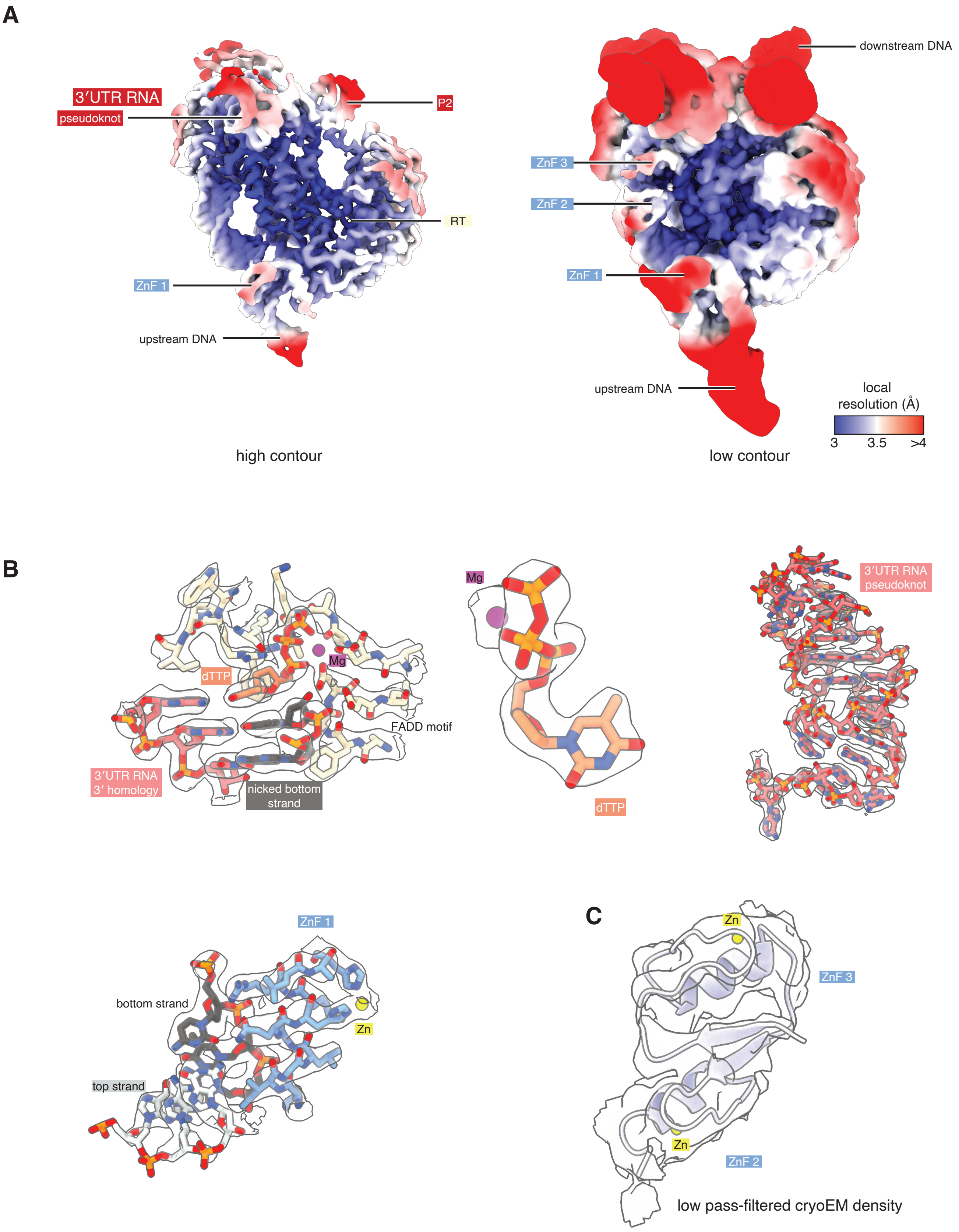

A
3′UTR RNA
pseudoknot
P2
RT
ZnF 1
upstream DNA
high contour
downstream DNA
ZnF 3
ZnF 2
ZnF 1
upstream DNA
low contour
local
resolution (Å)
3
3.5
>4
B
dTTP
Mg
FADD motif
3′UTR RNA
3′ homology
nicked bottom
strand
Mg
dTTP
3′UTR RNA
pseudoknot
ZnF 1
bottom strand
Zn
top strand
C
Zn
ZnF 3
Zn
ZnF 2
low pass-filtered cryoEM density

Supplementary Fig. 3: Additional R2Tg cryo-EM details.

A. Gaussian-filtered cryo-EM map coloured by local resolution as calculated using RELION, at two different contour levels.
B. Sharpened cryo-EM density for three different parts of the structure.
C. Low-pass filtered cryo-EM density for ZnF2 and ZnF3.

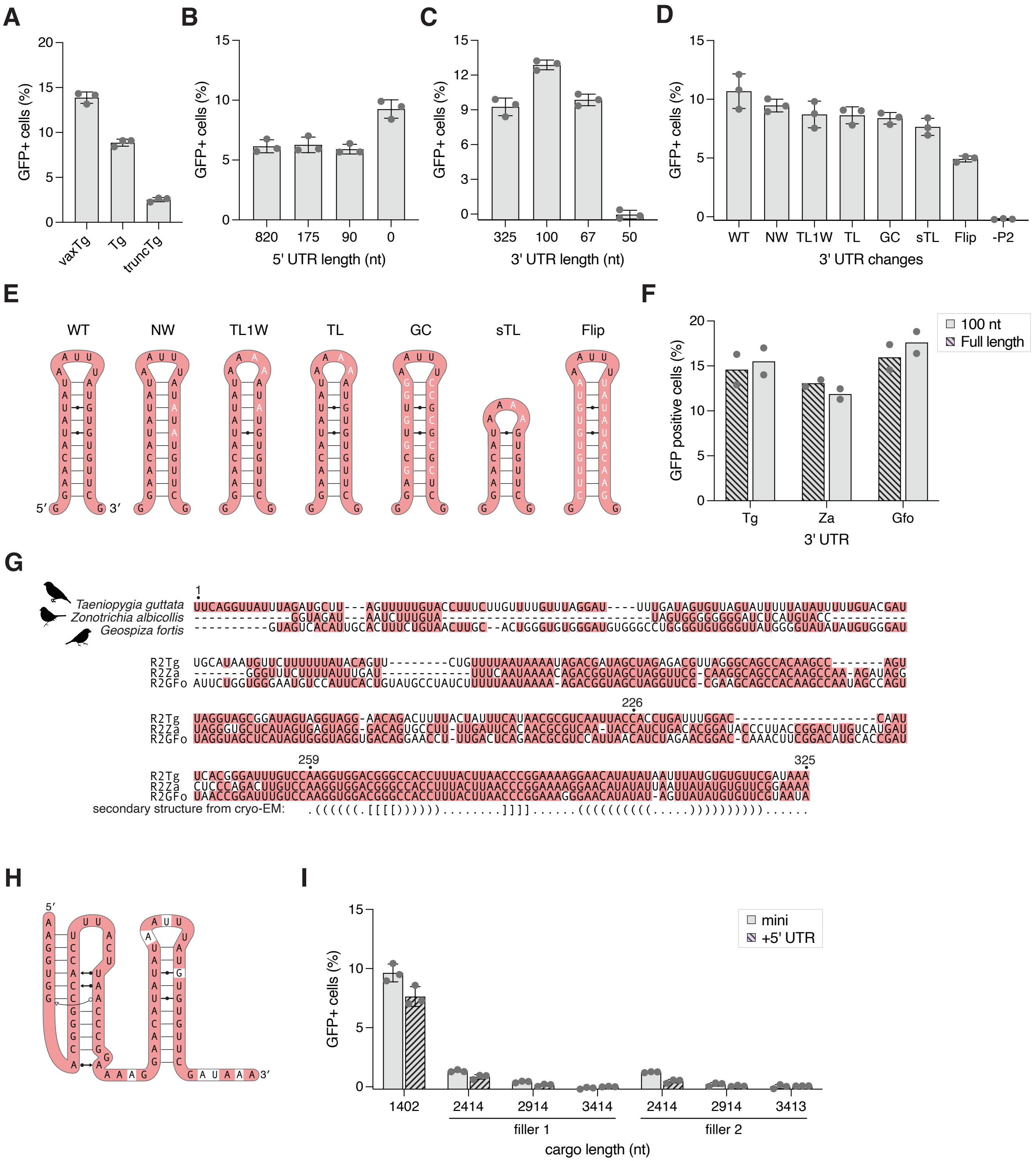

A
B
C
D
GFP+ cells (%)
vaxTg
Tg
truncTg
5' UTR length (nt)
820
175
90
0
3' UTR length (nt)
325
100
67
50
3' UTR changes
WT
NW
TL1W
TL
GC
sTL
Flip
-P2
E
F
GFP positive cells (%)
100 nt
Full length
Tg
Za
Gfo
3' UTR
G
Taeniopygia guttata
Zonotrichia albicollis
Geospiza fortis
R2Tg
R2Za
R2GFo
secondary structure from cryo-EM:
H
I
mini
+5' UTR
1402
2414
2914
3414
3413
filler 1
filler 2
cargo length (nt)

Supplementary Fig. 4: Additional characterization of R2Tg and donor requirements.

A. The integration efficiencies of the all-RNA system using different R2Tg mRNA (n = 3). vax-Tg, R2Tg mRNA with UTRs and poly(A) in Pfizer's BNT162b2 vaccine; Tg, R2Tg mRNA with 60 nt poly(A) tail and no functional UTRs; truncTg, R2Tg mRNA with 78 amino acid deletion on the N-terminus, 60 nt poly(A) tail, and no functional UTRs. Donor RNA with 5′ ribozyme from *Forficula auricularia* and 70 nt Tg 3′ UTR.
B. The effects of progressively truncating the donor's 5′ UTR from the 3′ direction, with an alternative way to normalize the RNA amounts between samples (n = 3). Each condition was transfected with the same total mass of RNA, with no fillers and 1:2 molar ratio of the editor and donor, respectively.
C. The effects of progressively truncating the donor's 3′ UTR from the 5′ direction, with an alternative way to normalize the RNA amounts between samples (n = 3). Each condition was transfected with the same total mass of RNA, with no fillers and 1:2 molar ratio of the editor and donor, respectively ("325" data in 4b as "0 nt 5′ UTR").
D. R2Tg's ability to integrate donors with 3′ UTRs that have different sequences but similar secondary structure (WT: wildtype, NW: no wobble base-pairs, TL1W: tetraloop with one wobble base-pair, TL: tetraloop, GC: GC-rich P2, sTL: tetraloop with short P2 stem, Flip: inverted nucleotides in the P2 stem, and -P2: deleted P2) (n = 3).
E. Predicted secondary structures of modified P2 described in 4d. White nucleotides highlight changes from the wildtype sequence.
F. R2Tg's ability to integrate donors with full length or 100 nt 3′ UTRs from Tg, Za, and Gfo (n = 2). Donor RNA with 5′ ribozyme from *Nasonia giraulti*. Editor mRNAs made with ψ.
G. Alignment between the 3′ UTRs of R2Tg, Za, and Gfo. Red background indicates identical nucleotides, and white background indicates differences between one or more orthologs.
H. Secondary structure diagram of R2Tg's 3′ UTR core, colored by sequence identity with R2Za and Gfo, as in 4g.
I. Effects of progressively increasing the cargo's length with two different filler sequences, with and without the full length 5′ UTR (n = 3). Each condition was transfected with 400 ng of total RNA with the same molar quantities of editor and donor (1:2 molar ratio). Filler RNA compensates for differences in total mass.

For bar graphs, each dot represents a technical replicate, the bars represent the mean, and the error bars represent the standard deviation. When relevant, p values are calculated with Student's two-sided t test. Source data are provided as a Source Data file.

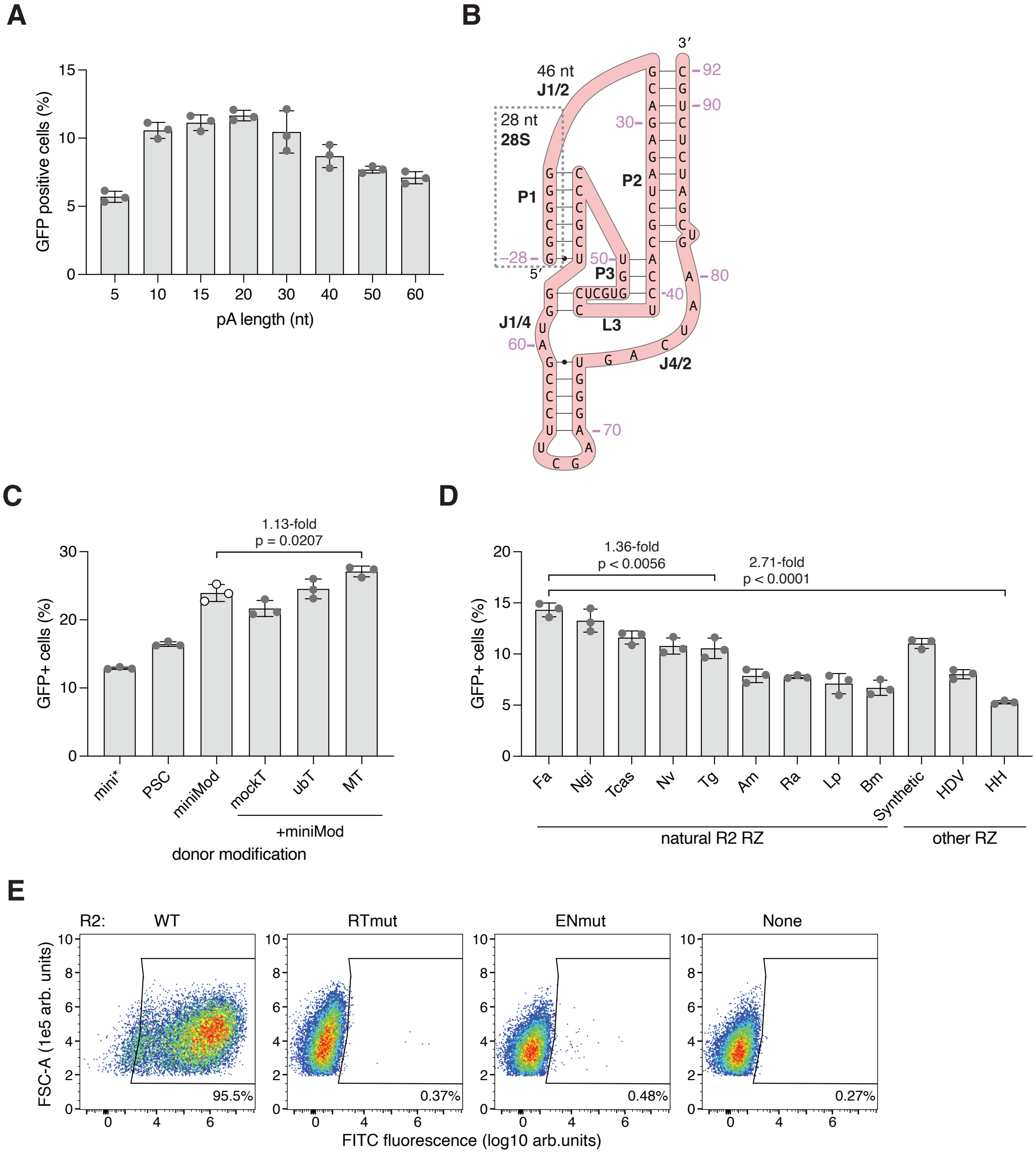

A
GFP positive cells (%)
15
10
5
0
5
10
15
20
30
40
50
60
pA length (nt)
B
3′
46 nt
J1/2
28 nt
28S
P1
P2
P3
L3
J1/4
J4/2
5′
-28
30
40
50
60
70
80
90
92
C
1.13-fold
p = 0.0207
GFP+ cells (%)
30
20
10
0
mini*
PSC
miniMod
mockT
ubT
MT
+miniMod
donor modification
D
1.36-fold
p < 0.0056
2.71-fold
p < 0.0001
GFP+ cells (%)
20
15
10
5
0
Fa
Ngi
Tcas
Nv
Tg
Am
Ra
Lp
Bm
Synthetic
HDV
HH
natural R2 RZ
other RZ
E
R2:
WT
RTmut
ENmut
None
FSC-A (1e5 arb. units)
10
8
6
4
2
0
4
6
95.5%
0.37%
0.48%
0.27%
FITC fluorescence (log10 arb.units)

Supplementary Fig. 5: Modifying donor RNA ends affect integration efficiency.

A. Effects of progressively increasing the donor RNA's poly(A) tail (n = 3).
B. Schematic of R2Tg's 5′ HDV-like ribozyme, which incorporates 28 nt of the 28S sequence upstream of its insertion site.
C. The additive effects of donor modifications (n = 3). PSC, 5′ modification with phosphorothioate bonds; mockT, miniMod with mock ligation for the 3′ tail; ubT, miniMod with unbranched modified poly(A) tail; MT, miniMod with branched and modified poly(A) tail.
D. The effects of varying the donor's 5′ ribozymes on integration efficiency (n = 3). Natural HDV-like ribozymes are derived from R2 elements from the following organisms: Fa, *Forficula auricularia*; Ngi, *Nasonia giraulti*; Tcas, *Tribolium castaneum*; Nv, *Nematostella vectensis*; Tg, *Taeniopygia guttata*; Am, *Anurida maritima*; Ra, *Rhynchosciara americana*; Lp, *Limulus polyphemus*; and Bm, *Bombyx mori*. Other tested ribozymes: synthetic, a sequence predicted to fold into an HDV-like ribozyme and incorporates 28S homology at the start of the ribozyme; HDV, ribozyme derived from hepatitis delta virus preceding 28S homology; HH, hammerhead ribozyme preceding 28S homology. Donor RNAs were made with ψ and Gfo 3′ UTR[21].
E. The resulting GFP signal when miniMod donors are cotransfected via LNP with wildtype R2Tg (WT), reverse transcriptase mutant (RTmut), endonuclease mutant (ENmut), or filler RNA (none) (data for one of the replicates in Fig. 4g).

For bar graphs, each dot represents a technical replicate, the bars represent the mean, and the error bars represent the standard deviation. When relevant, p values are calculated with Student's two-sided t test. Source data are provided as a Source Data file.

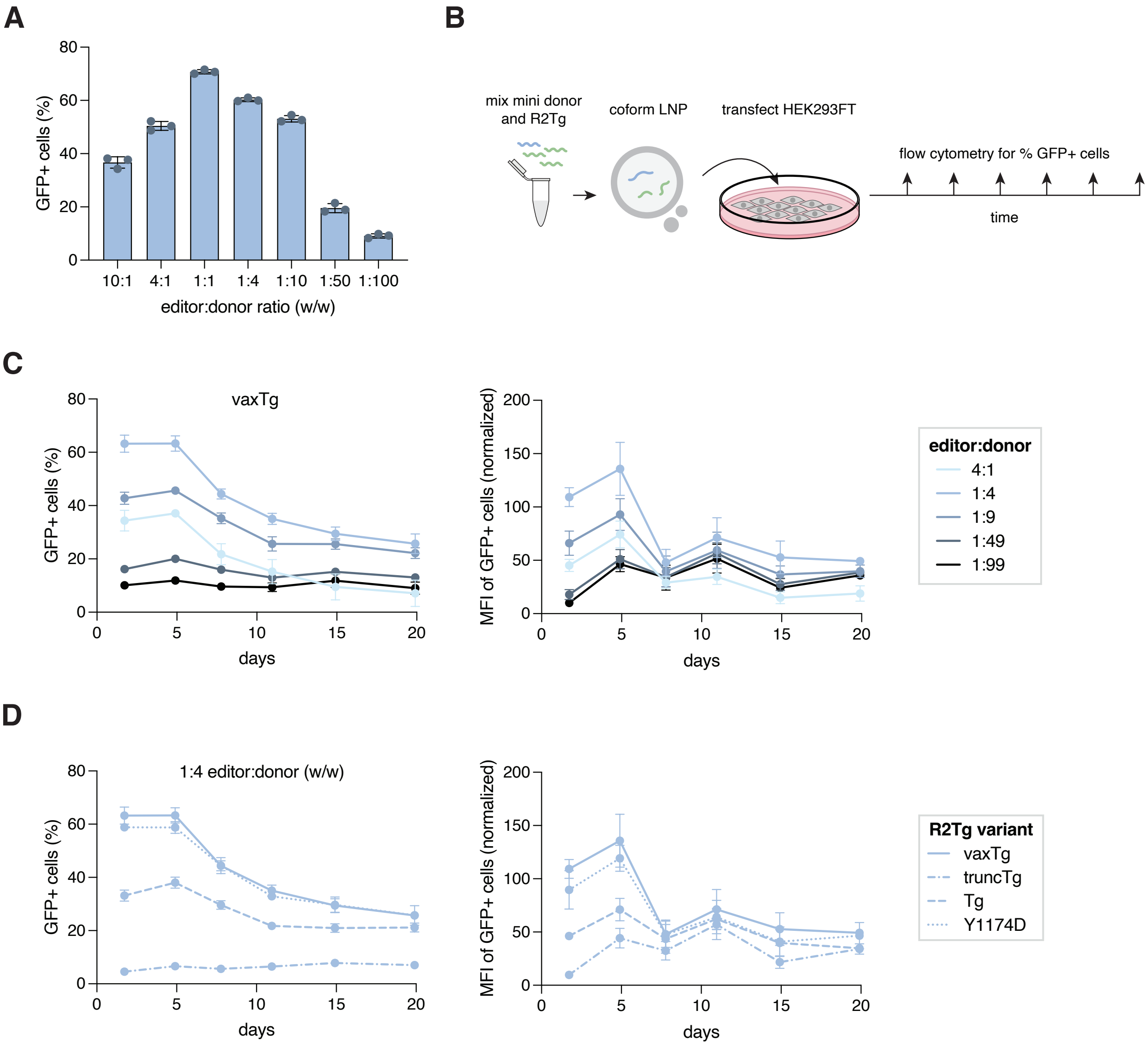

A
GFP+ cells (%)
80
60
40
20
0
10:1
4:1
1:1
1:4
1:10
1:50
1:100
editor:donor ratio (w/w)
B
mix mini donor and R2Tg
coform LNP
transfect HEK293FT
flow cytometry for % GFP+ cells
time
C
vaxTg
GFP+ cells (%)
days
MFI of GFP+ cells (normalized)
days
editor:donor
4:1
1:4
1:9
1:49
1:99
D
1:4 editor:donor (w/w)
GFP+ cells (%)
days
MFI of GFP+ cells (normalized)
days
R2Tg variant
vaxTg
truncTg
Tg
Y1174D

Supplementary Fig. 6: Following the LNP transfected population over time.

A. Effects of using different editor:donor ratios (w/w) on integration efficiency (n = 3). Mini donors and LNP delivery were used in this experiment. Each dot represents a technical replicate, the bars represent the mean, and the error bars represent the standard deviation.
B. Schematic of the experiment to follow the transfected cells over time. HEK293FT cells were transfected with LNPs co-formed with mini donor and R2Tg RNA.
C. The effects of using different R2Tg:donor ratios. Samples are transfected with the indicated ratios of editor:donor (w/w) and assayed for %GFP+ cells (left) or MFI of the GFP+ cells (right) over the course of approximately 20 days (n = 4, data presented as mean +/- standard deviation). %GFP+ cells are calculated after subtracting the background in samples transfected with 1:4 RTmut:donor. MFI of the GFP+ cells is normalized to the MFI of all single cells in the RTmut sample.
D. The effects of using different versions of R2Tg. Samples are transfected with the indicated R2Tg variant and assayed for %GFP+ cells (left) or MFI of the GFP+ cells (right) over the course of approximately 20 days (n = 4, data presented as mean +/- standard deviation). vaxTg, Tg, and truncTg are as described in Supplementary Fig. 4a. Y1174D is a mutant R2Tg mRNA. %GFP+ cells are calculated after subtracting the background in samples transfected with 1:4 RTmut:donor. MFI of the GFP+ cells is normalized to the MFI of all single cells in the RTmut sample. (“vaxTg” data in 6c as “1:4”).

Source data are provided as a Source Data file.

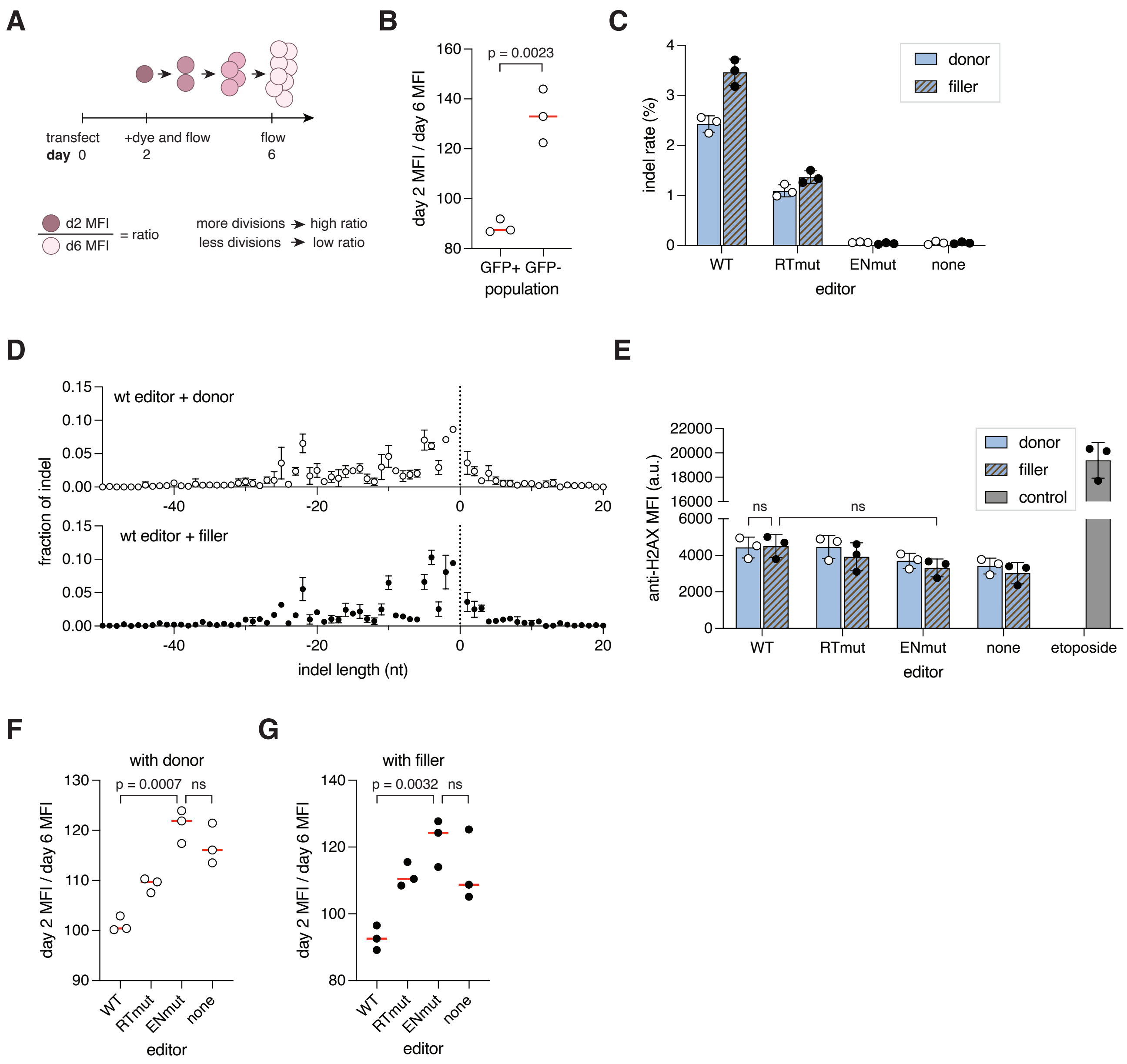
A
transfect
day 0
+dye and flow
2
flow
6
d2 MFI
d6 MFI
= ratio
more divisions → high ratio
less divisions → low ratio
B
p = 0.0023
day 2 MFI / day 6 MFI
GFP+ GFP-
population
C
indel rate (%)
donor
filler
WT
RTmut
ENmut
none
editor
D
wt editor + donor
wt editor + filler
fraction of indel
indel length (nt)
E
anti-H2AX MFI (a.u.)
donor
filler
control
ns
ns
WT
RTmut
ENmut
none
etoposide
editor
F
with donor
p = 0.0007
ns
day 2 MFI / day 6 MFI
WT
RTmut
ENmut
none
editor
G
with filler
p = 0.0032
ns
day 2 MFI / day 6 MFI
WT
RTmut
ENmut
none
editor

Supplementary Fig. 7. Effects of LNP-delivered R2Tg with and without donor RNA.

A. Schematic of the dye dilution assay to track cell proliferation. Unless otherwise specified, the MFI of the gated "single cell" population was used to calculate the d2/d6 ratio.
B. Proliferation of the GFP+ and GFP- populations ($n = 3$).
C. Indel rates generated by WT or mutant R2Tg, with and without donor RNA ($n = 3$).
D. Indel lengths generated by WT R2Tg with and without donor RNA within the -50 to 20 nt window ($n = 3$, data presented as mean +/- standard deviation).
E. γH2AX levels in response to R2Tg, as measured by anti-γH2AX MFI ($n = 3$). Cells treated with 50 μM of etoposide were used as a positive control.
F. Cell proliferation after transfecting WT or mutant R2Tg with donor RNA ($n = 3$).
G. Cell proliferation after transfecting WT or mutant R2Tg without donor RNA ($n = 3$).

Each experiment was performed with three technical replicates, bars represent the mean, and the error bars represent the standard deviation. When relevant, p values are calculated with Student's two-sided t test. Donor RNA is a GFP miniMod with 67-nt 3′ UTR. Filler RNA is a firefly luciferase mRNA. Source data are provided as a Source Data file.

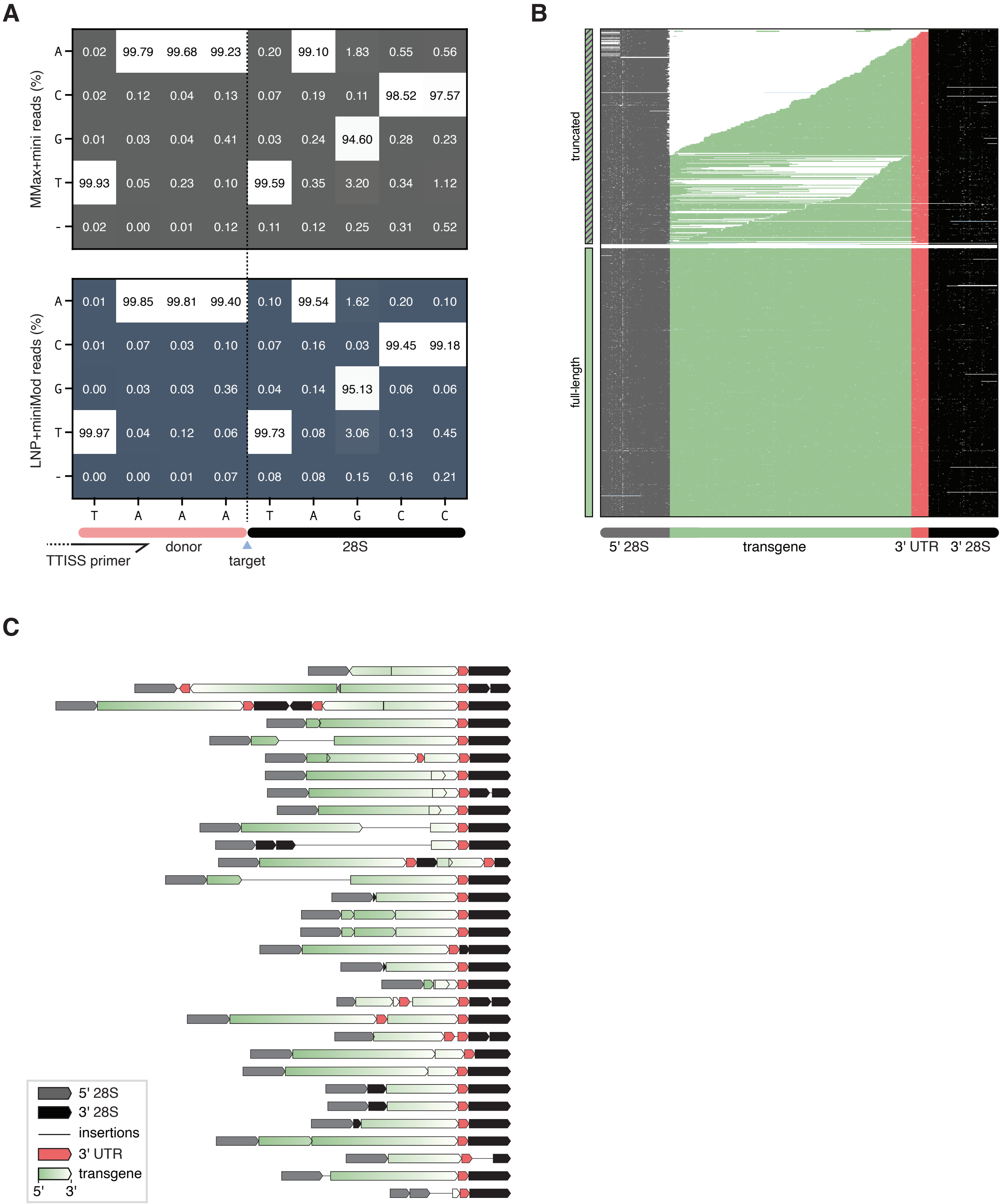
A
MMax+mini reads (%)
A 0.02 99.79 99.68 99.23 0.20 99.10 1.83 0.55 0.56
C 0.02 0.12 0.04 0.13 0.07 0.19 0.11 98.52 97.57
G 0.01 0.03 0.04 0.41 0.03 0.24 94.60 0.28 0.23
T 99.93 0.05 0.23 0.10 99.59 0.35 3.20 0.34 1.12
- 0.02 0.00 0.01 0.12 0.11 0.12 0.25 0.31 0.52
LNP+miniMod reads (%)
A 0.01 99.85 99.81 99.40 0.10 99.54 1.62 0.20 0.10
C 0.01 0.07 0.03 0.10 0.07 0.16 0.03 99.45 99.18
G 0.00 0.03 0.03 0.36 0.04 0.14 95.13 0.06 0.06
T 99.97 0.04 0.12 0.06 99.73 0.08 3.06 0.13 0.45
- 0.00 0.00 0.01 0.07 0.08 0.08 0.15 0.16 0.21
T A A A T A G C C
TTISS primer
donor
target
28S
B
truncated
full-length
5' 28S
transgene
3' UTR
3' 28S
C
5' 28S
3' 28S
insertions
3' UTR
transgene
5'
3'

Supplementary Fig. 8: Further characterization of the integration products.

A. The nucleotide identities at the 3′ insertion junction for MMax-delivered system with mini donor (top) and LNP-delivered system with miniMod donor (bottom) (n = 3, data presented as the mean of three technical replicates; source data are provided as a Source Data file).
B. Alignment of the full-length and truncated integration products from long-read sequencing. Reads are colored based on their alignment to the reference regions. Light blue represents mismatch or soft clips in the sequence alignments.
C. All "other" reads in Fig. 5c with feature annotations. Green color gradient indicates location of the read along the transgene.

A

name	organism	cell type
HSMM	human	primary myoblast
T cells	human	primary T
E14	mouse	embryonic stem
HepG2	human	hepatocellular carcinoma
BJ	human	fibroblast
STO	mouse	embryonic fibroblast
N2a	mouse	neuroblast
C2C12	mouse	myoblast
Huh7	human	hepatocellular carcinoma
AC16	human	cardiomyocyte
HeLa	human	cervical cancer
HEK293FT	human	embryonic kidney

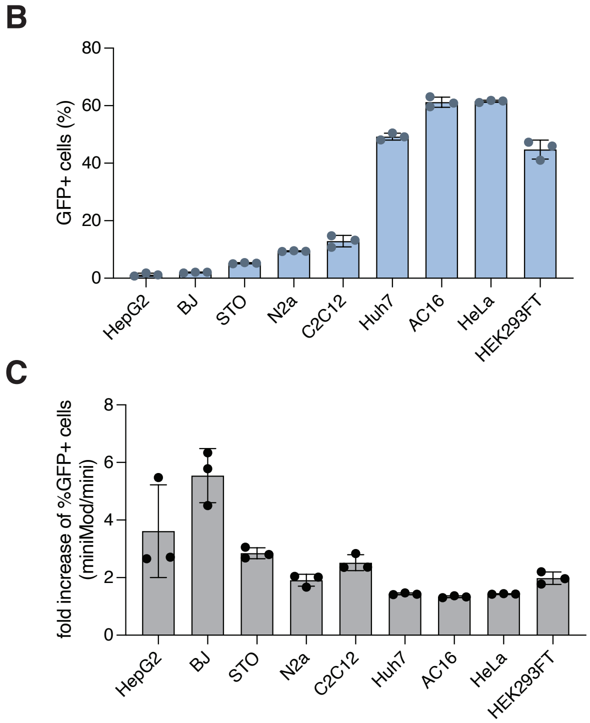

Supplementary Fig. 9: Mini donor and miniMod in diverse mammalian cell types.

A. The name, organism, and cell type of the cells described in Fig. 5.
B. The integration efficiency of our all-RNA system in different mammalian cell types, using LNP-delivered mini donor and R2Tg (n = 3).
C. The relative integration efficiency of mini donor vs. miniMod donor in different mammalian cell types (n = 3).

For bar graphs, each dot represents a technical replicate, the bars represent the mean, and the error bars represent the standard deviation. When relevant, p values are calculated with Student's two-sided t test. Source data are provided as a Source Data file.

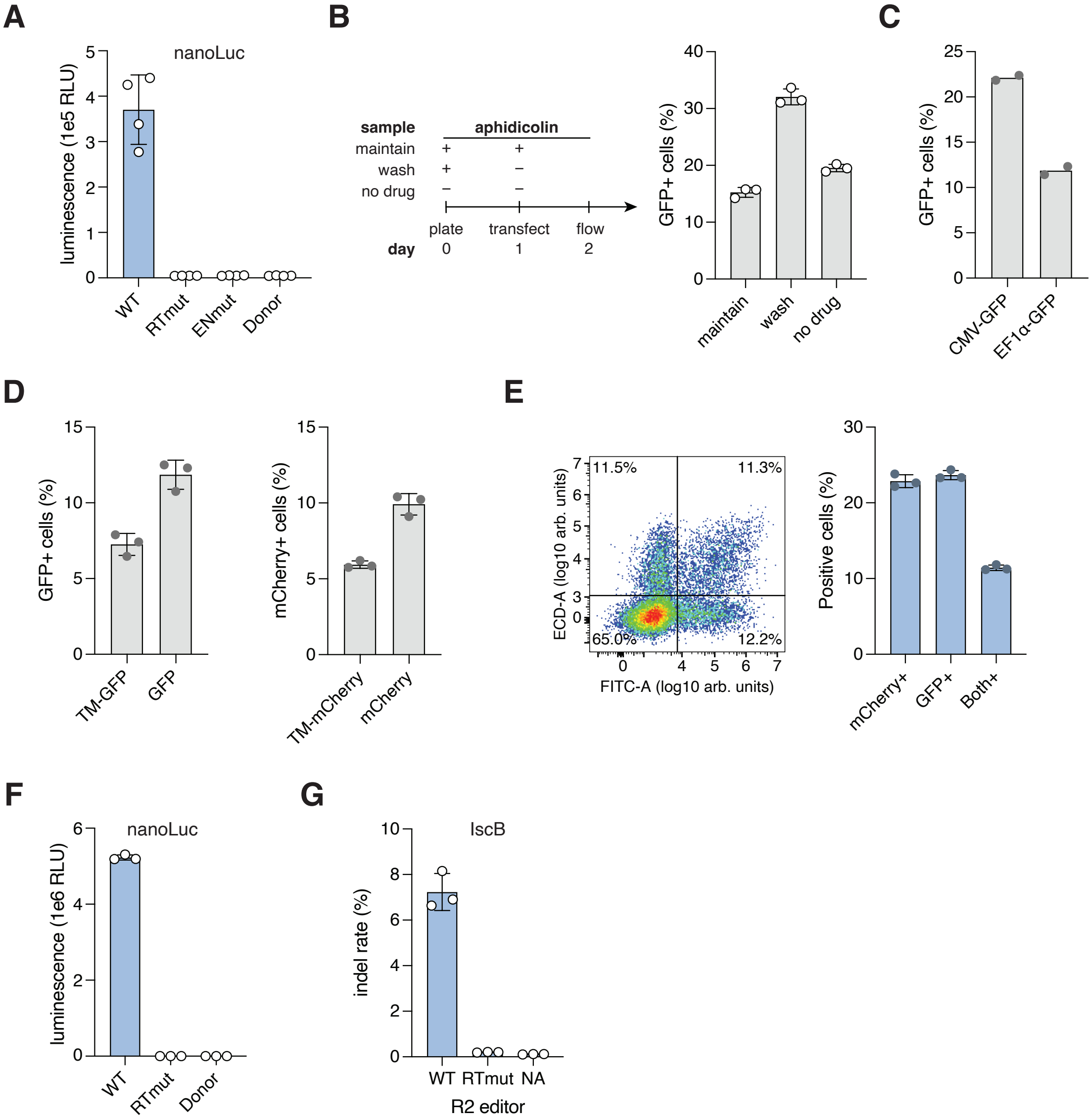
A
nanoLuc
luminescence (1e5 RLU)
WT
RTmut
ENmut
Donor
B
sample
aphidicolin
maintain
wash
no drug
plate
transfect
flow
day
GFP+ cells (%)
maintain
wash
no drug
C
GFP+ cells (%)
CMV-GFP
EF1α-GFP
D
GFP+ cells (%)
TM-GFP
GFP
mCherry+ cells (%)
TM-mCherry
mCherry
E
11.5%
11.3%
65.0%
12.2%
ECD-A (log10 arb. units)
FITC-A (log10 arb. units)
Positive cells (%)
mCherry+
GFP+
Both+
F
nanoLuc
luminescence (1e6 RLU)
WT
RTmut
Donor
G
IscB
indel rate (%)
WT
RTmut
NA
R2 editor

Supplementary Fig. 10: The system with different cell cycles and donor cargos.

A. Integrating nanoluciferase[63] miniMod donor (1243-nt cargo) in differentiated myotubes (*n* = 4). Luminescence was assayed approximately 44 hours after transfection.
B. Integration in aphidicolin treated HEK293FT cells (*n* = 3). Cells in the "maintain" and "wash" samples were treated with 100 ng/mL of aphidicolin diluted in complete media upon plating on a poly-D-lysine coated plate. 24 hours later, the cells were transfected with MMax-delivered miniMod system in media with ("maintain") or without ("wash" and "no drug") aphidicolin, alongside 20 ng of mCherry RNA as a cotransfection marker. One day later, the %GFP+ cells were analyzed by flow cytometry.
C. Testing donors with CMV and EF1α driven GFP as the cargo (1396- and 1973-nt cargo, respectively) (*n* = 2). HEK293FT were plated two days prior to transfection at 1e4 cells/well in a 96 well plate. Donor RNA with R2Fa ribozyme and 100-nt Gfo 3′ UTR.
D. Testing donors with GFP and transmembrane GFP (left) or mCherry and transmembrane mCherry (right) as the cargo (1402, 1669, 1405, and 1660-nt cargo, respectively) (*n* = 3).
E. Testing LNP-delivered system's ability to integrate multiple donors per cell. LNPs were co-formed with an R2Tg mRNA, GFP, and mCherry donors at a ratio of 1:2:2 (w/w). In the bar graph, %GFP+ and mCherry+ cells were background subtracted from untransfected samples (*n* = 3).
F. Testing donors with nanoluciferase reporter as the cargo (*n* = 3). Luminescence was assayed approximately 36 hours after transfection.
G. Functional integration of engineered IscB in HEK293FT cells (*n* = 3). Cells were transfected with LNP-delivered, engineered IscB miniMod (2683-nt cargo) and R2Tg. After expansion, they were transfected with ωRNA expression vector, then harvested for indel analysis 3 days later.

For bar graphs, each dot represents a technical replicate, the bars represent the mean, and when present, the error bars represent the standard deviation. Source data are provided as a Source Data file.

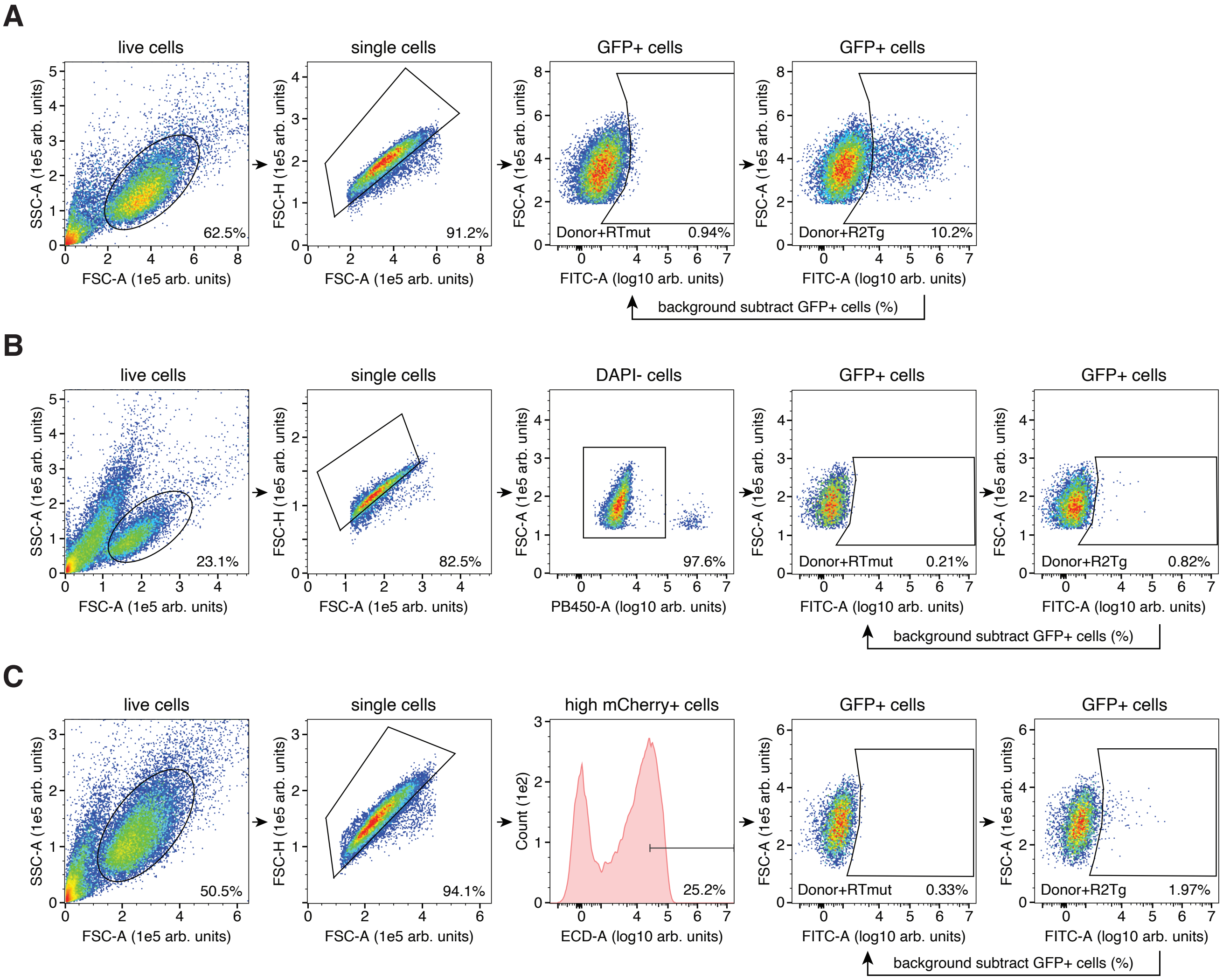
A
live cells
single cells
GFP+ cells
GFP+ cells
SSC-A (1e5 arb. units)
FSC-A (1e5 arb. units)
62.5%
FSC-H (1e5 arb. units)
91.2%
FSC-A (1e5 arb. units)
FITC-A (log10 arb. units)
Donor+RTmut
0.94%
Donor+R2Tg
10.2%
background subtract GFP+ cells (%)
B
live cells
single cells
DAPI- cells
GFP+ cells
GFP+ cells
23.1%
82.5%
PB450-A (log10 arb. units)
97.6%
Donor+RTmut
0.21%
Donor+R2Tg
0.82%
background subtract GFP+ cells (%)
C
live cells
single cells
high mCherry+ cells
GFP+ cells
GFP+ cells
50.5%
94.1%
Count (1e2)
ECD-A (log10 arb. units)
25.2%
Donor+RTmut
0.33%
Donor+R2Tg
1.97%
background subtract GFP+ cells (%)

Supplementary Fig. 11: Gating strategies for flow cytometry.

A. The basic gating strategy used to analyze %GFP+ cells. For each cell type, live cells were gated on SSC-A vs. FSC-A and single cells were gated on FSC-H vs. FSC-A. For each donor type and transfection method, GFP+ cells were gated on FSC-A vs. FITC-A. %GFP+ cells in samples transfected with donor+R2Tg are calculated by subtracting the background in either donor+RTmut or donor only samples. The example in this figure is from the experiment in Fig. 3d. This type of gating strategy was used in experiments described in Figs. 3c-h, 4a,b,d-i, 5f-h, and Supplementary Figs. 4a-d,f,i, 5a,c,d, 6a,c,d, 9b,c, and 10c,d. When relevant, the same principles were applied to donors using mCherry reporters.

B. The gating strategies used to analyze %GFP+ cells in primary human T cell experiment described in Fig. 5i. After gating for live and single cells and before gating for GFP+ cells, we gated for cells negative for DAPI signal as an additional screen against dead cells.

C. The gating strategies used to analyze %GFP+ cells in the "gated" population of E14 cells described in Fig. 5j. After gating for live and single cells and before gating for GFP+ cells, we gated for the top 25 percentile of mCherry (co-transfection marker) expressing cells. A similar strategy was used to gate for successfully transfected cells in the experiment described in Supplementary Fig. 10b, where we gated for mCherry+ (instead of a chosen percentile) population prior to GFP+ cells.
