# Supplementary material for: Structure and biochemistry-guided engineering of an all-RNA system for DNA insertion with R2 retrotransposons
Source: Nat Commun. 2025 Jul 2;16:6079. doi: 10.1038/s41467-025-61321-z (PMC12222951; doi:10.1038/s41467-025-61321-z)
Supplement: Supplementary file 2 — Description of Additional Supplementary Files [file 41467_2025_61321_MOESM2_ESM.pdf]

Description of Additional Supplementary Files

File name: Supplementary Data 1
Description: Plasmid, oligonucleotide, and crRNA sequences used in this study.

File name: Supplementary Data 2
Description: Cryo-EM data collection, refinement, and validation statistics.

File name: Supplementary Data 3
Description: Off-target loci detected by the TTISS experiment.
