# Supplementary material for: Structure and biochemistry-guided engineering of an all-RNA system for DNA insertion with R2 retrotransposons
Source: Nat Commun. 2025 Jul 2;16:6079. doi: 10.1038/s41467-025-61321-z (PMC12222951; doi:10.1038/s41467-025-61321-z)
Supplement: Supplementary file 6 — Reporting Summary [file 41467_2025_61321_MOESM6_ESM.pdf]

nature portfolio

Corresponding author(s): Feng Zhang

Last updated by author(s): May 14, 2025

Reporting Summary

Nature Portfolio wishes to improve the reproducibility of the work that we publish. This form provides structure for consistency and transparency in reporting. For further information on Nature Portfolio policies, see our Editorial Policies and the Editorial Policy Checklist.

Statistics

For all statistical analyses, confirm that the following items are present in the figure legend, table legend, main text, or Methods section.

n/a	Confirmed	
☐	☒	The exact sample size (*n*) for each experimental group/condition, given as a discrete number and unit of measurement
☐	☒	A statement on whether measurements were taken from distinct samples or whether the same sample was measured repeatedly
☐	☒	The statistical test(s) used AND whether they are one- or two-sided<br>*Only common tests should be described solely by name; describe more complex techniques in the Methods section.*
☒	☐	A description of all covariates tested
☒	☐	A description of any assumptions or corrections, such as tests of normality and adjustment for multiple comparisons
☐	☒	A full description of the statistical parameters including central tendency (e.g. means) or other basic estimates (e.g. regression coefficient) AND variation (e.g. standard deviation) or associated estimates of uncertainty (e.g. confidence intervals)
☐	☒	For null hypothesis testing, the test statistic (e.g. *F*, *t*, *r*) with confidence intervals, effect sizes, degrees of freedom and *P* value noted<br>*Give P values as exact values whenever suitable.*
☒	☐	For Bayesian analysis, information on the choice of priors and Markov chain Monte Carlo settings
☒	☐	For hierarchical and complex designs, identification of the appropriate level for tests and full reporting of outcomes
☒	☐	Estimates of effect sizes (e.g. Cohen's *d*, Pearson's *r*), indicating how they were calculated

Our web collection on statistics for biologists contains articles on many of the points above.

Software and code

Policy information about availability of computer code

Data collection	CytExpert (v2.5); MinION flow cell (v10.4.1)
Data analysis	RELION (v5.0); CTFFIND (v4.1); Topaz (v1.0); AlphaFold (v2.0); Coot (v9.8.1); ISOLDE (v1.7.1); ChimeraX (v1.7.1); PHENIX (v1.21.2-5419); GraphPad Prism (v10.4.2); FlowJo (v10.10.0); CRISPResso2 (v2.3.1); FIJI (ImageJ2 v2.14.0); SnapGene (v7.2.1); Geneious Prime (v2024.0.5); QuantaSoft (v1.7.4.0917); iTOL (v7.2); Dorado (v0.9.0); Custom code (https://doi.org/10.5281/zenodo.15414055)

For manuscripts utilizing custom algorithms or software that are central to the research but not yet described in published literature, software must be made available to editors and reviewers. We strongly encourage code deposition in a community repository (e.g. GitHub). See the Nature Portfolio guidelines for submitting code & software for further information.

Data

Policy information about availability of data

All manuscripts must include a data availability statement. This statement should provide the following information, where applicable:

- Accession codes, unique identifiers, or web links for publicly available datasets
- A description of any restrictions on data availability
- For clinical datasets or third party data, please ensure that the statement adheres to our policy

The structure of R2Tg is available on PBD (accession 9DOU), and the cryo-EM map is available on EM databank (code EMD-47091). TTISS and long-read sequencing

results are available on SRA (BioProject PRJNA1163071). All plasmid, oligonucleotide, and crRNA sequences are detailed in Supplementary Data 1. Source data are provided with this paper.

Research involving human participants, their data, or biological material

Policy information about studies with human participants or human data. See also policy information about sex, gender (identity/presentation), and sexual orientation and race, ethnicity and racism.

Reporting on sex and gender	No human research participants
Reporting on race, ethnicity, or other socially relevant groupings	No human research participants
Population characteristics	No human research participants
Recruitment	No human research participants
Ethics oversight	No human research participants

Note that full information on the approval of the study protocol must also be provided in the manuscript.

Field-specific reporting

Please select the one below that is the best fit for your research. If you are not sure, read the appropriate sections before making your selection.

☒ Life sciences ☐ Behavioural & social sciences ☐ Ecological, evolutionary & environmental sciences

For a reference copy of the document with all sections, see nature.com/documents/nr-reporting-summary-flat.pdf

Life sciences study design

All studies must disclose on these points even when the disclosure is negative.

Sample size	All experiments in mammalian cells were performed with at least 3 replicates to ensure reproducibility, with the exception of the long-read sequencing experiment, and experiments described in Supplementary Figs. 4f and 10c.
Data exclusions	No data was excluded.
Replication	All attempts at replication were successful.
Randomization	Randomization is not relevant to the study. It was not required for the types of experiments performed.
Blinding	Blinding was not used. Quantifications were done via software and calculations.

Reporting for specific materials, systems and methods

We require information from authors about some types of materials, experimental systems and methods used in many studies. Here, indicate whether each material, system or method listed is relevant to your study. If you are not sure if a list item applies to your research, read the appropriate section before selecting a response.

Materials & experimental systems

n/a	Involved in the study
☐	☒ Antibodies
☐	☒ Eukaryotic cell lines
☒	☐ Palaeontology and archaeology
☒	☐ Animals and other organisms
☒	☐ Clinical data
☒	☐ Dual use research of concern
☒	☐ Plants

Methods

n/a	Involved in the study
☒	☐ ChIP-seq
☐	☒ Flow cytometry
☒	☐ MRI-based neuroimaging

Antibodies

Antibodies used	αCD3 (Biolegend 300328; Clone HIT3a; Lot B347319; used at 1:50) αCD25 (Biolegend 302617; Clone BC96; Lot B348548; used at

Antibodies used	1:50); αH2AX (BD Biosciences 560447; Clone N1-431; Lot 4176446; used at 1:10); LaG-41 αGFP-nanobody (this study, described in Methods)
Validation	αCD3 (Biolegend 300328) and αCD25 (Biolegend 302617): Each lot of these antibodies is quality control tested by immunofluorescent staining with flow cytometric analysis by BioLegend; αH2AX (BD Biosciences 560447): BD Biosciences confirmed specificity through multiple methodologies.

Eukaryotic cell lines

Policy information about cell lines and Sex and Gender in Research

Cell line source(s)	HEK293FT (ThermoFisher, R70007); HepG2 (ATCC, HB-8065); E14 (ATCC, CRL-1821); N2a (ATCC, CCL-131); C2C12 (ATCC, CRL1772); STO (ATCC, CRL1503); BJ (ATCC, CRL-2522); HeLa (gift from Paul Blainey lab); HSMM (Lonza, CC-2580); Huh7 (Broad Genetic Perturbation Platform); AC16 (Sigma, SCC109)
Authentication	Cell lines had expected morphology and approximate doubling times. They were not additionally authenticated.
Mycoplasma contamination	Cell lines were not further tested for mycoplasma contamination after purchase.
Commonly misidentified lines (See ICLAC register)	Cell lines were not listed in ICLAC register.

Plants

Seed stocks	No plants were used in this study.
Novel plant genotypes	No plants were used in this study.
Authentication	No plants were used in this study.

Flow Cytometry

Plots

Confirm that:

☒ The axis labels state the marker and fluorochrome used (e.g. CD4-FITC).

☒ The axis scales are clearly visible. Include numbers along axes only for bottom left plot of group (a 'group' is an analysis of identical markers).

☒ All plots are contour plots with outliers or pseudocolor plots.

☒ A numerical value for number of cells or percentage (with statistics) is provided.

Methodology

Sample preparation	Sample preparation is described in detail in the Method. Adherent cells were washed once with PBS (Gibco) and detached with TrypLE Express (Gibco) at 37C. Cells were then resuspended in FACS buffer made from 0.5% BSA (LGC Clinical Diagnostics) and 5 mM EDTA (Invitrogen) diluted in PBS. Resuspended cells were then transferred to non-treated V-bottom plates for flow cytometry.<br>Suspension cells (T cells) were centrifuged for 500 g for 5 minutes. Culture media was then replaced with Flow Buffer made from 1% FBS and 2 mM EDTA diluted in PBS. When relevant, cells were stained with antibodies for 45 minutes, then washed twice with Flow Buffer. 100 ng/mL DAPI diluted in Flow Buffer was used for the final wash. Cells were then resuspended in Flow Buffer prior to flow cytometry.
Instrument	CytoFLEX S (Beckman Coulter)
Software	Data collection: CytExpert (v2.5)<br>Data analysis: FlowJo (v10.10.0); GraphPad Prism (v10.4.2)
Cell population abundance	Three days after primary T cell activation, cells were stained with αCD25. Flow cytometry confirmed that more than 99% were CD25+. Purchased cell lines were used for all other experiments.
Gating strategy	Gating strategies are described in detail in Extended Data Fig. 11. For each cell type, live cells were gated by FSC-A and SSC-A and single cells were gated by FSC-A and FSC-H. For each donor and transfection method, GFP+ cells were gated by FSC-A and FITC-A.

In T cell experiments, cells were stained with DAPI as an additional way to distinguish between live and dead cells. Live, DAPI negative cells were gated by FSC-A and PB450-A, after gating for single cells and before gating for GFP+ cells.
In E14 and aphidicolin experiments, cells were additionally gated on mCherry cotransfection marker by FSC-A and ECD-A, after gating for single cells and before gating for GFP+ cells.

☒ Tick this box to confirm that a figure exemplifying the gating strategy is provided in the Supplementary Information.
